# Metabolic rates of the Asian shore crab *Hemigrapsus sanguineus* in air as a function of body size, location, and injury

**DOI:** 10.1002/ece3.9297

**Published:** 2022-09-09

**Authors:** Laura S. Fletcher, Mikayla Bolander, Tanner C. Reese, Emily Gail Asay, Emily Pinkston, Blaine D. Griffen

**Affiliations:** ^1^ Department of Biology Brigham Young University Provo Utah USA

**Keywords:** energetics, invasive species, metabolism, non‐lethal injury

## Abstract

Rapid warming in the Gulf of Maine may influence the success or invasiveness of the Asian shore crab, *Hemigrapsus sanguineus*. To better predict the effects of climate change on this invasive species, it is necessary to measure its energy dynamics under a range of conditions. However, previous research has only focused on the metabolism of this intertidal species in water. We sampled adult crabs from three different sites and measured their metabolic rates in the air. We show that metabolic rate increases with body mass and the number of missing limbs, but decreases with the number of regenerating limbs, possibly reflecting the timing of energy allocation to limb regeneration. Importantly, metabolic rates measured here in the air are ~4× higher than metabolic rates previously measured for this species in water. Our results provide baseline measurements of aerial metabolic rates across body sizes, which may be affected by climate change. With a better understanding of respiration in *H. sanguineus*, we can make more informed predictions about the combined effects of climate change and invasive species on the northeast coasts of North America.

## Graphical abstract

The Asian shore crab, Hemigrapsus sanguineus, is an invasive species along the northeast coast of North America. Previous work has focused on metabolic rate measurements for this species in water, but not in air. Our study examined the metabolic rate of this invasive species in air as a function of body size, location, and injury.

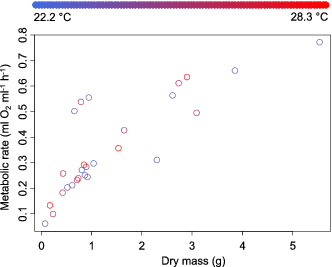

## 1 INTRODUCTION

Energetic adaptations often give invasive species a competitive edge over their native counterparts (Cooper & Eme, 2020; da Silva et al., 2021; Lagos, Barneche, et al., 2017; Lagos, White, et al., 2017). Some of these adaptations include a broad thermal tolerance (Cooper & Eme, 2020; da Silva et al., 2021), decreased metabolic rate during reproduction (da Silva et al., 2021), increased tolerance to hypoxic conditions (Lagos, Barneche, et al., 2017), and high mass‐specific metabolic rates (Lagos, White, et al., 2017). Such traits underpin the success of many invasive species and may make them more resilient to climate change than native competitors (da Silva et al., 2021). Studying the energetics of invasive species can therefore help to explain their invasive success, and whether this success will continue under changing conditions brought about by climate change.

Rapid warming of northern seas makes the northeast coast of the United States a prime focal area for the study of invasive species and climate change (Belkin, 2009). Since the 1950s, temperatures in the Gulf of Maine alone have risen by three times the average rate of global sea surface warming, making it one of the fastest‐warming marine areas in the world (Pershing et al., 2015). Rising temperatures have increased the area's susceptibility to novel and ongoing biological invasions, including that of the Asian shore crab (*Hemigrapsus sanguineus*).

Native to the Western Pacific, *H. sanguineus* was first introduced to the coast of New Jersey in 1988 (Blakeslee et al., 2017; Williams & McDermott, 1990) and quickly spread, outcompeting native species and previously established invasive species such as the European green crab (*Carcinus maenas*) (Griffen et al., 2021; Kraemer et al., 2007). Numerically, *H. sanguineus* has become the dominant crab species throughout its invaded range (Lord & Williams, 2017), and has depressed populations of crab competitors and prey species (Kraemer et al., 2007). Several reasons for its success have been proposed, including the ability to outcompete native species for food and shelter (Griffen et al., 2008, 2011; Jensen et al., 2002; Lohrer & Whitlatch, 2002a), high fecundity (Fukui, 1988), direct predation on native and other invasive crab species (Lohrer & Whitlatch, 2002b), and the absence of natural parasites and predators in invaded regions (Blakeslee et al., 2009; McDermott, 2011). Although it prefers animal prey in lab choice experiments (Brousseau & Baglivo, 2005; Griffen et al., 2012, 2015), *H. sanguineus* is a generalist omnivore capable of consuming other crabs, bivalves, and algae (Bourdeau & O'Connor, 2003; Gerard et al., 1999), and can tolerate wide ranges of temperature and salinity (Gerard et al., 1999).

Recent evidence suggests that energetic traits may also play a role in the success of *H. sanguineus*. Limb loss is highly prevalent in this species, and at any given time, >40% of individuals may be missing one or more limbs (Davis et al., 2005). However, despite such a high incidence of limb loss, its energetic state (i.e., stored energy in the hepatopancreas) appears to be mostly unaffected by injury (Vernier & Griffen, 2019). While this could simply reflect efficient energy extraction from food (Jungblut et al., 2018), it may also imply some degree of energetic resilience to injury, possibly related to an altered metabolic rate during limb regrowth or a reduction in the metabolic cost of recovery. Such resilience would leave more energy for reproduction, foraging, and other activities, potentially allowing this species to outcompete native competitors. However, little research has been done on the metabolic rate of *H. sanguineus* and on whether it changes in response to injury.

Metabolic rate is a direct measurement of an organism's energy use. Previous work comparing *H. sanguineus* and *C. maenas* metabolism in water suggests a promising connection between metabolic rate and invasion success (Jungblut et al., 2018). Jungblut et al. (2018) demonstrated that *H. sanguineus* consumes oxygen at nearly double the rate than *C. maenas* does at 20°C, resulting in higher activity and a competitive edge when foraging in water at high temperatures. However, *H. sanguineus* is an upper intertidal species that spends a significant amount of time in the air each day (Epifanio, 2013). The respiratory anatomy of a closely related congener, *Hemigrapsus nudus*, reveals adaptations for frequent aerial exposure (Greenaway et al., 1996). These species have modified gill lamellae to facilitate aerial respiration, and the branchial chambers' inner walls are modified to form rudimentary lungs (Greenaway & Farrelly, 1990). Overall, the gills of *Hemigrapsus* species are much smaller and their lungs are much more developed than purely aquatic species (Greenaway et al., 1996). Therefore, a complete understanding of *H. sanguineus* metabolism requires measuring respiration in the air as well as in water.

Quantifying the metabolism of *H. sanguineus* is especially important given the rapidly changing climate in the Gulf of Maine (Pershing et al., 2015). Climate change is predicted to accelerate the propagation and success of some invasive species and decrease the success of others (Hellmann et al., 2008). Poikilothermic invaders in particular may benefit from rising temperatures associated with climate change because growth and metabolism typically increase with temperature among poikilotherms, resulting in heightened activity, foraging, and reproduction (Jungblut et al., 2018; Parry, 1983). However, depending on the magnitude of climate change in a given region, invasive poikilotherm fitness could be negatively impacted. Temperatures near the upper limits of a poikilotherm's thermal tolerance cause metabolism to increase more rapidly than consumption, producing a mismatch between metabolic needs and resource intake (Rall et al., 2010) and a reduction in ingestion efficiency (Lemoine & Burkepile, 2012), likely leading to lower fitness. The effects of climate change on the success of *H. sanguineus* will therefore depend on its physiology. However, before we can predict the effects of climate change on this species, we must gain a more complete understanding of its energetics, particularly during periods of aerial exposure.

To improve our understanding of energetics in *H. sanguineus*, we measured the metabolic rates of males and females in the air at three different sites throughout the northern portion of *H. sanguineus*' range. We expect that metabolic rate will increase with body size, will increase with temperature as expected for poikilotherms, and will increase with the degree of injury (number of missing limbs) reflecting the metabolic cost of recovery. These metabolic rates, combined with previous measurements in water (Jungblut et al., 2018), will provide a more complete understanding of *H. sanguineus*' respiration and the influence of size, temperature, and injury. Such key ecological information will lead to more informed predictions about the role energetics play in the success of this invasive poikilotherm throughout its invaded range.

## 2 METHODS

### 2.1 Crab collections and metabolic rate measurements

All sampling took place between October 12 and 15, 2021. We haphazardly sampled a total of 215 individuals by hand along the shores of Cape May Ferry, New Jersey (coordinates: 38°58′5.02″N 74°57′43.12″W; *n* = 75), Goshen Point in Waterford, Connecticut (coordinates: 41°17′56.1″N 72°06′44.9″W; *n* = 65), and Odiorne Point, New Hampshire (43° 2′13.34″N 70°42′57.42″W; *n* = 75). We stored crabs in a plastic container with approximately 1 cm of seawater to prevent desiccation until the crabs could be used in trials that same day.

We measured the metabolic rate of each individual under field conditions out of direct sunlight (since this species generally takes shelter under boulders during low tide) by measuring oxygen consumption with a constant volume technique (e.g., Leighton, 2018). For experimental chambers, we used 150‐ml plastic syringes sealed shut at the tips with silicone and with an 8‐mm port drilled in the syringe barrel that was used for extracting a gas sample. We placed each crab inside the barrel of the syringe and adjusted the plunger to vary chamber volume based on the size of the crab (see below). Before sealing the syringe, we measured the barometric pressure, ambient temperature, and relative humidity with a BTMeter (Model 100‐AAP). After placing crabs in the chambers, we allowed a 5‐min acclimation period, after which we sealed the chamber by placing a septum designed for use in headspace gas analysis (Bridge Analyzers Incorporated, model #001620) over the port in the chamber barrel. We recorded the start and end times for each trial to allow calculation of the trial duration for each crab.

We tested most individuals alone. However, very small crabs (~10 mm CW or smaller) were tested in groups of 2–4 in the same chamber to ensure measurable changes in oxygen concentration. We expected larger crabs to consume more oxygen, and therefore adjusted the volume of the chamber so that larger crabs had greater volume. In general, we used chamber volumes of 70–100 ml for crabs ~20 mm CW or greater and 40–70 ml for crabs <20 mm CW.

Similarly, the amount of time each crab spent in the chamber varied based on the size of the crab. We left smaller crabs in the chambers for longer amounts of time to ensure that a measurable change in oxygen concentration took place. The time period, therefore, varied between 20 and 120 min. Chamber volumes and trial duration were determined by the size of the crab and, based on preliminary trials, were chosen to ensure measurable but minimal changes in oxygen concentration during each trial. Variations in volume and trial duration were accounted for in the calculations of metabolic rate for each crab (see below). We initially measured the activity level of crabs inside experimental chambers by performing scan samples each minute during a trial to determine whether or not each crab was active. However, experimental crabs remained nearly motionless inside chambers throughout each trial. Our results, therefore, reflect resting metabolic rates. Each crab was tested only once.

At the end of each trial, we measured the final partial concentration of oxygen in the chamber by inserting a needle through the sampling port that was connected to a multi‐gas oxygen probe from Forensics Detectors™ (Model # FD‐600, 0.01% resolution) and withdrawing a gas sample at a rate of 0.5 L min^−1^ using the built‐in pump. Oxygen levels in experimental chambers always remained well above levels, which cause problems due to anoxia for air‐breathing crustaceans (Schmitz & Harrison, 2004). Specifically, the minimum final O_2_ level encountered was 19.19%, with mean final levels at 2.11% ± 0.22%. Similarly, CO_2_ levels in experimental chambers always remained well below hypercapnia levels, which cause problems for crabs (Burnett, 1997). Specifically, the maximum final CO_2_ level encountered was 1.45%, with mean final levels at 0.62% ± 0.18%. Following each trial, crabs were stored in individual plastic bags, frozen immediately on dry ice, transported to Brigham Young University in Provo, Utah, and stored at −80°C until dissection.

### 2.2 Dissections

For each crab, we determined the sex, measured the wet mass, and counted the number of missing limbs and the presence of any regenerating limb buds. We also determined whether each female was vitellogenic by removing the ovaries via dorsal dissection and visually inspecting them for egg development under a dissecting microscope. None of the individuals we used for analysis were vitellogenic or gravid at the time of collection. We then dried each crab to constant weight at 60°C and measured their dry mass to 0.0001 g using a Mettler Toledo DualRange scale (Model number XS205).

### 2.3 Metabolic rate calculations

We calculated oxygen consumption using equation 4.4 from Leighton (2018):

$$\mathrm{Vol}O_{2}=\frac{\left[V\left(F_{i}O_{2}-F_{e}O_{2}\right)-F_{e}O_{2}\left(\mathrm{Vol}H_{2}O\right)\right]}{\left[1-F_{e}O_{2}\left(1–\mathrm{RQ}\right)\right]\;}$$

where $\mathrm{Vol}O_{2}$ is the volume of oxygen consumed, V is the volume of the gas in the chamber, $F_{i}O_{2}$ is the initial fractional concentration of oxygen within the chamber (i.e., atmospheric oxygen, 0.2094), $F_{e}O_{2}$ is the final fractional concentration of oxygen within the chamber, $\mathrm{Vol}H_{2}O$ is the change in the volume of water vapor in the chamber, and RQ is the respiratory quotient, represented by a ratio of CO_2_ production to oxygen consumption that is between 0.7 and 1.0 (Leighton, 2018). For our calculations, we assumed an intermediate RQ value of 0.85. This is an appropriate assumption given the fact that *H. sanguineus* is omnivorous (Bourdeau & O'Connor, 2003; Gerard et al., 1999) and this RQ value minimizes the possible error in metabolic rate at 3% (Vleck, 1987). Additionally, by including a small amount of water in each syringe (<1 ml), we ensured that the volume of water vapor would remain relatively constant at saturation, yielding a change in water vapor volume ($\mathrm{Vol}H_{2}O$) of 0. We calculated the volume of gas in the chamber, V, by subtracting the volume of the crab from the volume of the chamber as set for each crab individually. To estimate the volume of the crab, we divided wet mass by the density of *H. sanguineus* to calculate an estimated body volume for the crab. We assumed a density of 1.1 g cm^−3^ given the fact that crabs sink in water, which has a density of 1.0 g cm^−3^. Any error associated with this assumption is small due to the small size of the crabs relative to the chambers.

Finally, we converted the volume of oxygen consumed ($\mathrm{Vol}O_{2}$) to metabolic rate ($\mathrm{Vol}O_{2}$ h^−1^) by dividing the trial duration for each individual crab in minutes and multiplying this value by 60. We also accounted for any chambers with multiple crabs by dividing the overall rate of oxygen consumption by the number of crabs in each chamber, yielding a per crab rate of hourly oxygen consumption. We then adjusted this metabolic rate based on standard temperature and pressure using equation 2.1 from Leighton (2018).

### 2.4 Statistical analyses

We analyzed our data with linear models using R v.4.1.2. We initially analyzed metabolic rates with data on males and females and all sites pooled together; however, due to a significant interaction between body mass and gender and between temperature and site, we decided to analyze each sex at each site separately using linear models. Predictor variables included dry body mass (g), the number of missing limbs, the number of regenerating limbs, and the ambient temperature at the time of metabolic rate measurements (°C), with the metabolic rate (ml O_2_ h^−1^) as our response variable. The exception to this was the analysis for Connecticut where we did not include temperature because the range of temperatures throughout the experimental trials was only 0.5°C. For each individual analysis, we first ran the full model, including all interactions, and then used the step function in the base R package to identify the best‐fitting model based on AIC. We report only the best‐fitting models for each gender at each site.

## 3 RESULTS

When all the data across sexes and sites were pooled and analyzed together, we found significant interactions between the effects of body size and sex (*t* = −2.02, *p* = .045) and between temperature and collection site (*t* = 3.10, *p* = .002). We, therefore, analyzed the data separately for each sex at each site.

### 3.1 New Jersey

**FIGURE 1:**
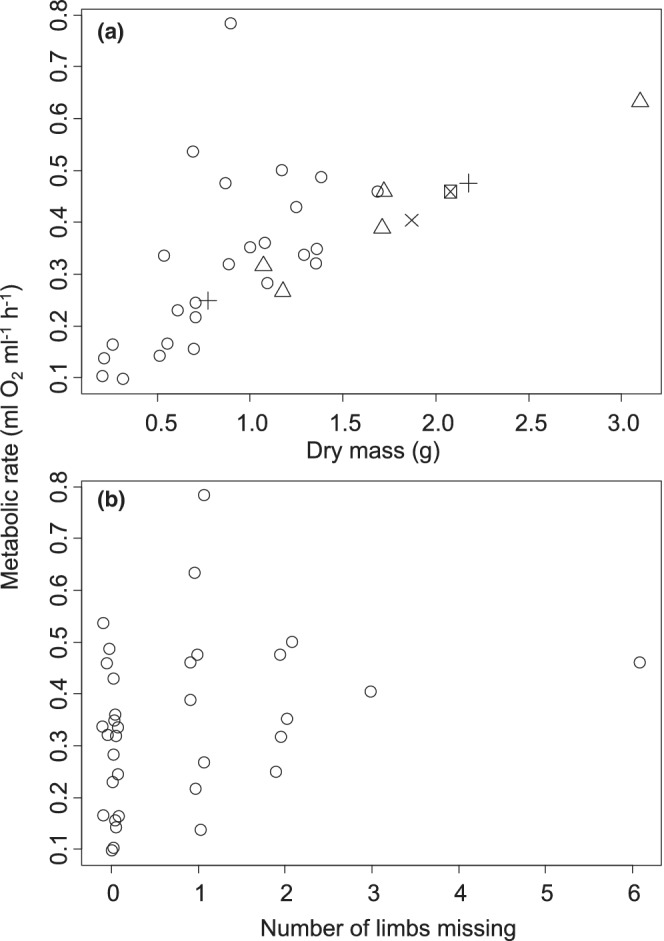
Metabolic rate for New Jersey *Hemigrapsus sanguineus* females as a function of body mass (a) and number of missing limbs (b). Each data point represents an individual. The shape of each datapoint in part A designates the number of regenerating limbs: a circle represents zero regenerating limbs, a triangle represents one regenerating limb, a + represents two regenerating limbs, an *x* represents three regenerating limbs, and a square represents four regenerating limbs. Data jittered along the *x*‐axis of part B for clarity of presentation.

For females, metabolic rate increased by 0.191 ± 0.037 ml O_2_ h^−1^ for every additional gram of dry body mass (*t* = 5.163, *p* < .0001, Figure 1a) and 0.076 ± 0.036 ml O_2_ h^−1^ with each additional missing limb (*t* = 2.148, *p* = .040, Figure 1b). On the other hand, metabolic rate decreased by −0.095 ± 0.038 ml O_2_ h^−1^ for each regenerating limb (*t* = −2.459, *p* = .020, Figure 1a). No other factors were included in the best‐fitting model.

**FIGURE 2:**
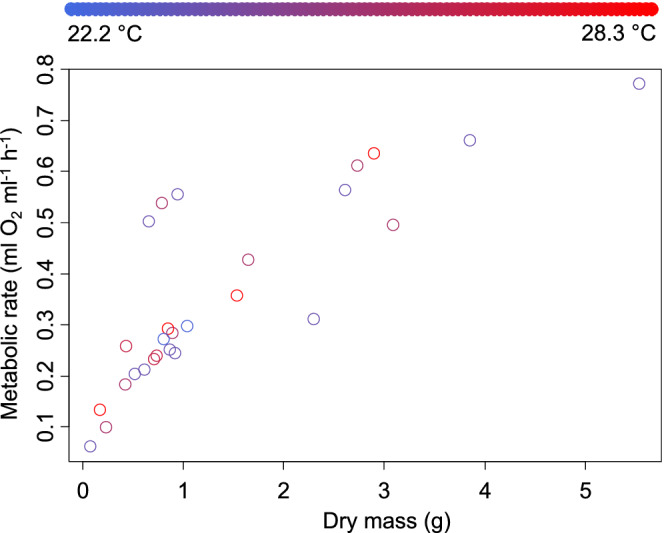
Metabolic rate for New Jersey *Hemigrapsus sanguineus* males as a function of body mass (*x*‐axis) and experimental temperature (symbol color using scale across top of figure).

For males, the metabolic rate also increased with dry body mass, at a rate of 1.106 ± 0.276 ml O_2_ h^−1^ for each additional gram of body mass (*t* = 4.004, *p* = .0006, Figure 2). Male oxygen consumption increased with temperature at a rate of 0.082 ± 0.015 ml O_2_ h^−1^ for each rise of 1°C (*t* = 5.567, *p* = <.0001). The interaction between temperature and body mass was significant as well (*t* = −3.682, *p* = .001). No other factors were included in the best‐fitting model.

### 3.2 Connecticut

**FIGURE 3:**
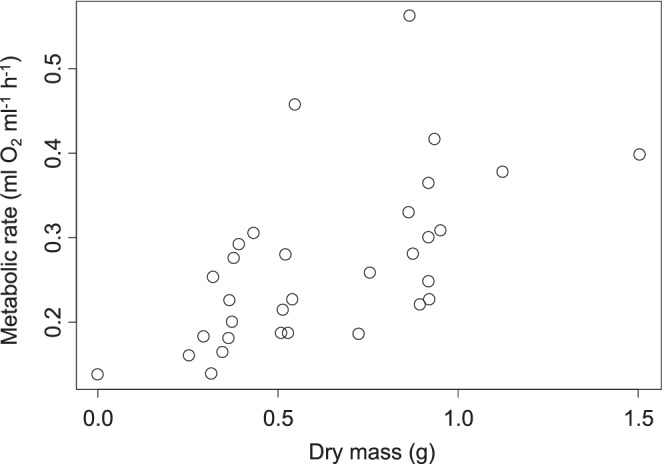
Metabolic rate for Connecticut *Hemigrapsus sanguineus* females as a function of body mass. Each data point represents an individual.

Female oxygen consumption increased by 0.184 ± 0.031 ml O_2_ h^−1^ for every additional gram of dry body mass (*t* = 4.121, *p* = .0003, Figure 3). No other factors were included in the best‐fitting model.

**FIGURE 4:**
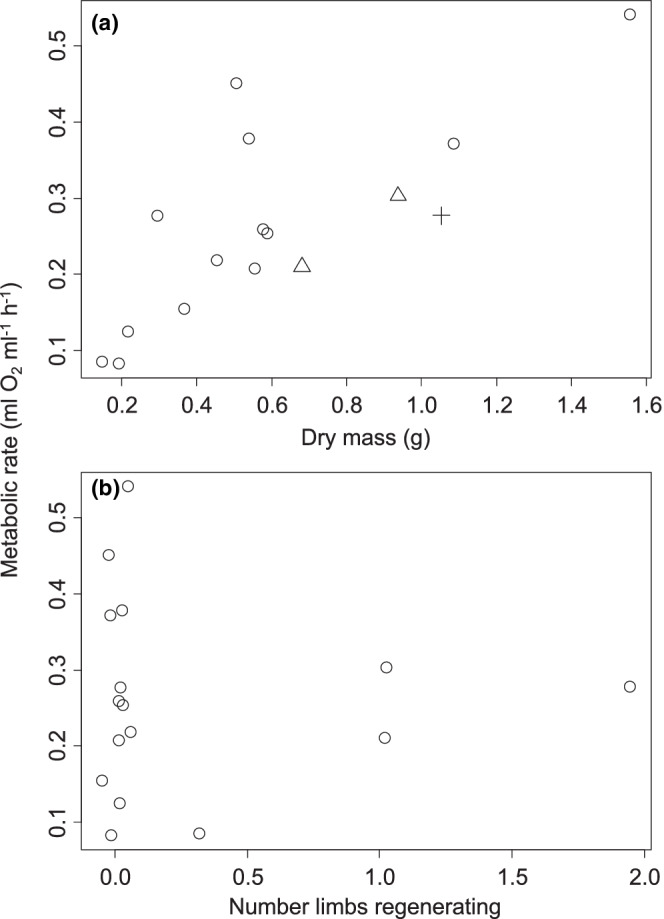
Metabolic rate for Connecticut *Hemigrapsus sanguineus* males as a function of body mass (a) and the number of limbs that are regenerating (b). Each data point represents an individual with the exception of the single data point showing 0.5 legs regenerating in part B, in which case this shows the average of two crabs that were too small to measure independently, one that was missing a limb and the other that was not. Data in part B jittered along the *x*‐axis for clarity of presentation.

Males experienced an increase in metabolic rate of 0.324 ± 0.061 ml O_2_ h^−1^ for every additional gram of body mass (*t* = 5.345, *p* < .001, Figure 4) and a decrease of 0.101 ± 0.042 ml O_2_ h^−1^ with each additional missing limb that was being regenerated (*t* = −2.415, *p* = .033). The number of missing limbs was included in the best‐fitting model but was not significant (*p* = .204). No other factors were included in the best‐fitting model.

### 3.3 New Hampshire

**FIGURE 5:**
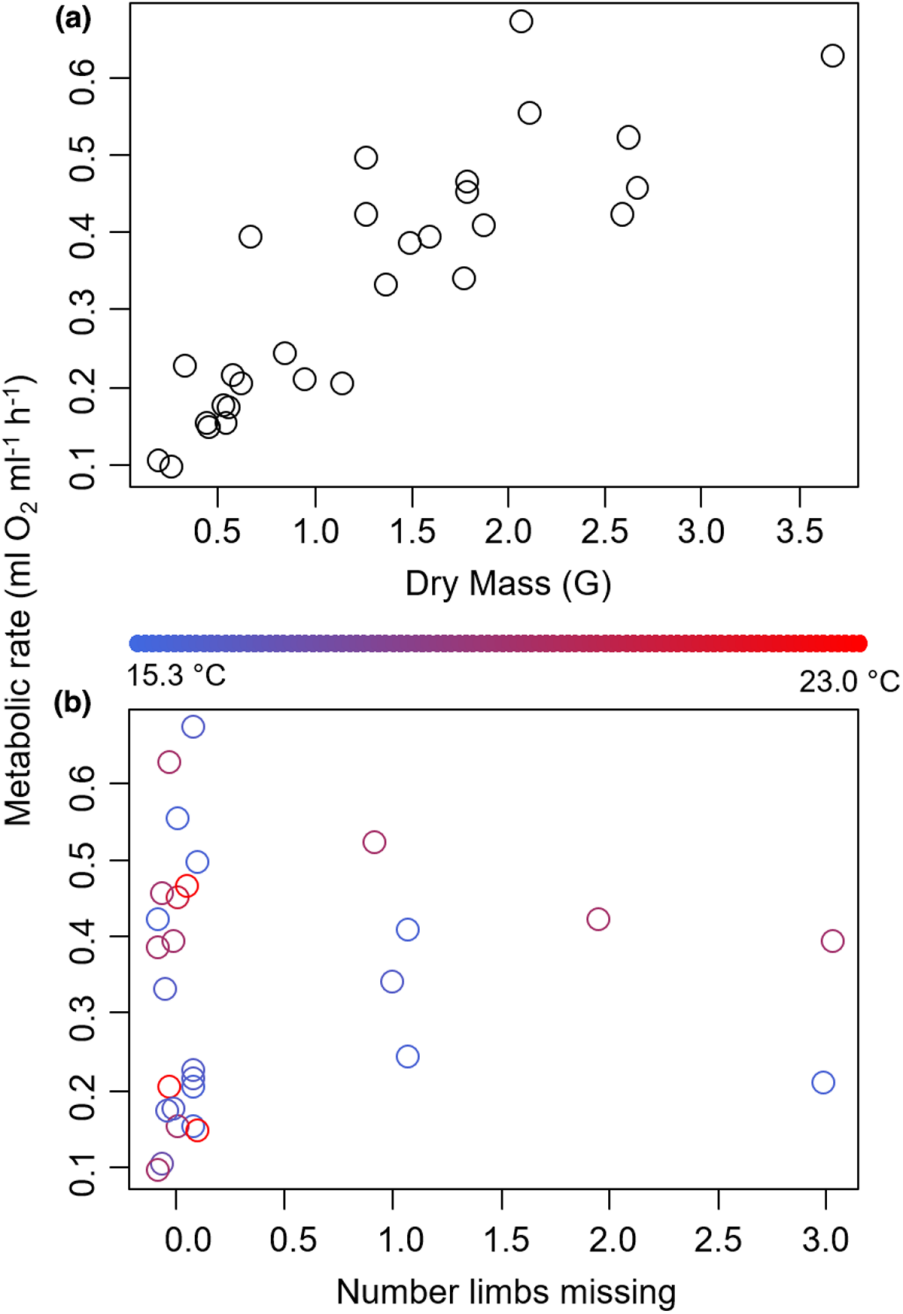
Metabolic rate for New Hampshire *Hemigrapsus sanguineus* females as a function of body mass (a) and the number of missing limbs (b). Each data point represents an individual. Data in part B are jittered for clarity of presentation.

For females, metabolic rate increased by 0.172 ± 0.125 ml O_2_ h^−1^ for every additional gram of body mass (*t* = 9.351, *p* < .0001, Figure 5). Female metabolic rate also increased by 0.479 ± 0.196 ml O_2_ h^−1^ for each additional missing limb (*t* = 2.445, *p* = .022). The interaction between temperature and the number of missing limbs was also significant (*t* = −2.458, *p* = .022). The main effect of temperature was therefore included in the best‐fitting model but was not significant (*p* = .227).

**FIGURE 6:**
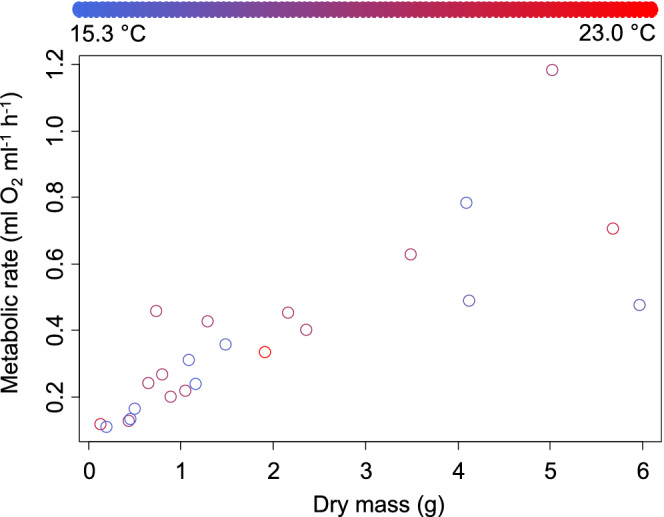
Metabolic rate for New Hampshire *Hemigrapsus sanguineus* males as a function of body mass (*x*‐axis) and experimental temperature (symbol color). Each data point represents an individual.

Metabolic rate in males was influenced by body mass (*t* = −2.341, *p* = .030) and by the interaction between temperature and body mass (*t* = 2.88, *p* = .010, Figure 6). No other factors were included in the best‐fitting model.

## 4 DISCUSSION

As expected, we found that the metabolic rate of *H. sanguineus* was influenced by body size and somewhat by temperature. We also found that metabolic rates were at times influenced by injury, generally increasing with the number of limbs that were missing, but tended to decrease with the number of missing limbs that were regenerating.

Jungblut et al. (2018) reported that the metabolic rate for *H. sanguineus* in water increased with both body size and temperature. We identified similar trends in the air; however, metabolic rates in the air were approximately four times higher than those reported by Jungblut et al. (2018) in water. To make a direct comparison, we used WebPlotDigitizer (https://automeris.io/WebPlotDigitizer/) to capture the data for metabolism as a function of body mass at 20°C from Figure 2 of Jungblut et al. (2018) and converted their wet body masses to dry masses by multiplying by 0.26, the ratio of dry to wet mass for crustaceans (Ricciardi & Bourget, 1998). Using a linear relationship fit to their data, a crab with a dry body mass of 2 g at 20°C should have a metabolic rate in the water of 0.111 ml O_2_ h^−1^. By comparison, using the statistical relationship for female crabs at our New Hampshire site, a 2‐g crab would have a metabolic rate in the air of 0.477 ml O_2_ h^−1^. The precise reason for this difference is unknown, but it could reflect differences in the time since feeding (24 h in Jungblut et al. (2018) and unknown in our study) and/or fundamental differences in the rate of energy expenditure in air vs. water. Regardless, we believe it would be valuable to measure both aerial and aquatic metabolic rates concurrently in an experimental setting.

We hypothesized that the metabolic rate would increase with temperature in air, just as it does in water (Jungblut et al., 2018). However, a relatively narrow range of temperatures at each site at the time of sampling (New Hampshire: 15–21°C; Connecticut: 21–22°C; and New Jersey: 22–28°C) limited the importance of temperature in our analyses. While metabolism did increase with temperature at the New Jersey site, we did not detect the same correlation at the other sites. To document a more complete physiological response to temperature in the air, future research should focus on *H. sanguineus* respiration under a broader range of temperatures, and could potentially use laboratory‐controlled temperatures similar to measurements in water (Jungblut et al., 2018) to facilitate direct comparisons. In addition, identifying a specific Q_10_ for *H. sanguineus* respiration in the air will improve our ability to make predictions about its physiological response to warming temperatures and its capacity to invade new areas.

We hypothesized that the metabolic rate would increase with the number of missing limbs, and our results support this hypothesis. An increase in metabolic rate following recent limb loss may reflect the beginning of the limb regeneration process. Prior to the appearance of limb buds, injured crabs allocate energy toward protein synthesis to generate the limb bud, causing an increase in basal metabolic rate (Hopkins & Das, 2015). Our measurements therefore likely captured the early costs of regeneration before the appearance of limb buds. This cost of regeneration may have implications for reproduction and survival if too many limbs are lost. In harvestmen (*Nelima paessleri*), a significant increase in metabolic rate only occurs after the loss of three or more limbs (Escalante et al., 2021). In contrast, our results show that metabolic rates increase linearly with the number of missing limbs in *H. sanguineus*.

Contrary to our hypothesis, the number of regenerating limbs was negatively correlated with metabolic rate. This could reflect the timing of energy allocation to limb regrowth. In injured crabs, there are two major periods of energy allocation (and increased metabolic rate). The first, as described above, occurs immediately following limb loss as the bud is produced, and the second takes place right before molting (Hopkins & Das, 2015). The presence of limb buds indicates that our measurements took place after the injured crabs passed the first period of energy allocation to produce limb buds, but before the second period of energy allocation associated with molting. Thus, for crabs with existing limb buds, our measurements may not have captured the metabolic cost of regeneration. It is not clear why metabolism decreased with limb buds present compared to crabs with all their limbs, although it could reflect a reduction in energy allocation to non‐regenerative metabolic processes, such as growth and reproduction. Whatever the reason, a decrease in metabolism associated with the presence of limb buds may also contribute to the energetic resilience to injury observed by Vernier and Griffen (2019). Our findings illustrate the need to uncover the exact timing of metabolic rate adjustments throughout limb regeneration and determine the reason for the decrease in metabolic rate between periods of energy allocation and whether this decrease is adaptive.

The need to more deeply understand metabolic adaptations associated with limb loss—and other metabolic adaptations—is especially pressing considering the rapid warming in the Gulf of Maine (Pershing et al., 2015). Metabolic injury resilience and other energetic traits may be altered by rising temperatures and elevated metabolic rates associated with climate change (Parry, 1983). Climate change may also lead to smaller adult body sizes in crustaceans such as *H. sanguineus* (Jaramillo et al., 2017), which would in turn alter metabolism. While crabs with smaller body sizes typically consume less total oxygen than larger conspecifics, they exhibit higher mass‐specific oxygen consumption rates (Leffler, 1972), which could change the amount of energy available for limb regeneration and other activities. Higher temperatures and elevated metabolic rates also lead to a reduction in the aerobic scope of an individual, or the excess energy available after maintenance costs have been met, significantly reducing reproduction, growth, and other activities vital to an organism's fitness (Pörtner et al., 2017). Through changes to metabolic rate and associated energetic traits, climate change may therefore have implications for the future invasive success of *H. sanguineus* in the Gulf of Maine.

In conclusion, our results show how *H. sanguineus* metabolism scales with body size and the degree of injury. We provide the first aerial metabolic rate measurements for *H. sanguineus*, which are approximately four times higher than those previously measured in water. With these measurements, our study provides a baseline for understanding low‐tide metabolism by *H. sanguineus* throughout the northern part of its invaded range. Combined with an understanding of high‐tide metabolism obtained from water measurements, we can use this baseline to make more direct comparisons to native and invasive competitors and explore the influence of metabolism on invasive success. The factors examined here, including body size and injury, may also be affected by climate change. Our measurements may therefore be used to improve predictions about the invasive success of *H. sanguineus* as climate change occurs along the northeast coast of North America.

## AUTHOR CONTRIBUTIONS

**Laura S. Fletcher:** Formal analysis (supporting); investigation (equal); visualization (lead); writing – original draft (lead); writing – review and editing (equal). **Mikayla Bolander:** Investigation (equal); writing – review and editing (equal). **Tanner C. Reese:** Investigation (equal); writing – review and editing (equal). **Emily Gail Asay:** Investigation (equal); writing – review and editing (equal). **Emily Pinkston:** Investigation (equal); writing – review and editing (equal). **Blaine D. Griffen:** Conceptualization (lead); formal analysis (lead); funding acquisition (lead); investigation (equal); methodology (lead); writing – original draft (supporting); writing – review and editing (equal).

## CONFLICT OF INTEREST

None declared.

## Data Availability

All data from this paper have been deposited in Dryad at https://doi.org/10.5061/dryad.msbcc2g20.
